# Pattern of Statin Use in Southern Italian Primary Care: Can Prescription Databases Be Used for Monitoring Long-Term Adherence to the Treatment?

**DOI:** 10.1371/journal.pone.0102146

**Published:** 2014-07-29

**Authors:** Carmen Ferrajolo, Vincenzo Arcoraci, Maria Giuseppa Sullo, Concetta Rafaniello, Liberata Sportiello, Rosarita Ferrara, Angelo Cannata, Claudia Pagliaro, Michele Giuseppe Tari, Achille Patrizio Caputi, Francesco Rossi, Gianluca Trifirò, Annalisa Capuano

**Affiliations:** 1 Campania Regional Centre of Pharmacovigilance and Pharmacoepidemiology, Experimental Medicine Department, Pharmacology Section, Second University of Naples, Naples, Italy; 2 Medical Informatics Department, Erasmus University Medical Centre, Rotterdam, Netherlands; 3 Clinical and Experimental Medicine Department, University of Messina, Messina, Italy; 4 Local Health Unit of Caserta, Caserta, Italy; University of Milan, Italy

## Abstract

**Objectives:**

We sought to evaluate the prescribing pattern of statins according to national and regional health policy interventions and to assess specifically the adherence to the therapy in outpatient setting in Southern Italy.

**Methods:**

A population-based study was performed on persons ≥15 years old, living in the catchment area of Caserta (Southern Italy), and registered in Arianna database between 2004 and 2010. Prevalence and incidence of new treatments with statins were calculated for each year and stratified by drug. Adherence to therapy was measured by Medication Possession Ratio. Sub-analyses by individual compound and type of cardiovascular prevention were performed.

**Results:**

From 2004 to 2010, the one-year prevalence of statin use increased from 44.9/1,000 inhabitants to 79.8/1,000, respectively, consistently with the incidence of new use from 16.2/1,000 to 19.5/1,000, except a slight decrease after criteria reimbursement revision on 2005 (13.3/1,000). The incidence of new treatments decreased for atorvastatin, and increased for simvastatin over the study years. Overall, 43% of new users were still highly adherent to the treatment (MPR≥80%) after six months, while 26% after 4-years of follow-up. As compared with highly adherent patients, the probability to be non-adherent (MPR≤25%) at 4-years of follow-up was 26% higher for women than for men (full adj. odds ratio: 1.26; 95% CI: 1.10–1.45), and 64% higher in patients who started on primary rather than on secondary prevention (1.64; 1.29–2.07).

**Conclusions:**

Prevalence and incidence of statin use increased consistently with health policy interventions. Only one-fourth of patients who newly initiated a statin were adherent to the treatment after 4-year of follow-up. Since the benefits of statins in terms of cardiovascular outcome and costs are associated with their chronic use, the identification of patient-related predictors of non-adherence such as gender, primary prevention could be suitable for physicians to improve the patients' compliance.

## Introduction

Cardiovascular diseases (CVDs) represent one of the primary cause of mortality in Western countries, accounting for 54% of all deaths in women and 43% of all deaths in men [1]. The role of hypercholesterolemia as cardiovascular risk factor is well-recognised, thus the introduction of lipid-lowering treatment with statins has represented one of the most important achievement of the recent medicine. In the last decade, the use of statins is widely increased in both United States and European countries because of the large body of evidence demonstrating their effectiveness in the cardiovascular prevention, both in subjects with or without history of CVD [2]. In particular, between 2003 and 2011, the use of these drugs showed a positive trend in Italy, representing also, in the last year of this period, the drug class with the highest expenditure (16.4 euro per capita) in Italy [3]. To optimize the use of lipid-lowering drugs, in 2004 the Italian Medicine Agency (*Agenzia Italiana del Farmaco*, AIFA) revised the reimbursement criteria (NOTA 13), according to which the purchase of statins was reimbursed by the National Health Service either for primary prevention in patients with familial hypercholesterolemia/hyperlipidaemia, those with 10-years risk of CVD >20% (based on national risk charts) or for secondary prevention, in subjects with previous well-established cardiovascular or cerebrovascular events, including patients affected by diabetes mellitus [4], [5]. As a consequence of the above national health policy intervention, in Campania region, in November 2008 a regional health policy intervention encouraging the prescription of two statins with expired patent, simvastatin or pravastatin, in new users with high cardiovascular risk was issued. A secondary aim of this intervention was to promote the adherence to the treatment with the ultimate goal to fully achieve the benefits of statin therapy [6].

In spite of the increased use of statins worldwide, recent findings documented the poor adherence to these drugs in the general population [7]–[10]. Adherence to medications has been associated with improved outcomes in coronary artery disease [11]. Further evidence suggested that, in general, improved adherence to a number of chronic disease medications, e.g. antidiabetic drugs, may also reduce overall healthcare costs [12]. The importance of adherence to statin therapy for primary or secondary prevention is well-documented. To date, reliable predictors of non-adherence to statin treatment have not clearly established [13]. Since very low adherence to treatment may reduce protective effects of statins, it could be useful to readily recognise such predictors to improve the level of adherence and consequently the effectiveness of statin treatment [14].

For this reason, we explored the healthcare record database from Local Health Unit of Caserta, in Campania region (Southern Italy), to describe the prescription pattern of statins in primary care setting, in relation to national and regional health policy interventions. Then, we assessed statin-specific rate and predictors of poor adherence to treatment in the same population.

## Materials and Methods

### Data source

This retrospective, drug utilization study was performed retrieving data from the Arianna database during the years 2004–2010. The database, which was set up by the Local Health Service of Caserta in the year 2000, currently contains information on almost 400,000 individuals living in the catchment area of Caserta and registered on the lists of 289 GPs. Collected information included patients' demographic characteristics and drug prescription without charge classified according the Anatomical Therapeutic Chemical (ATC) classification system, linked to medical diagnoses coded by the International Classification of Disease, 9^th^ revision, Clinical Modification (ICD-9). This database allowed us to anonymously track clinical and drug history routinely collected for each patient. All GPs enrolled in the Arianna database had previously been trained in data collection techniques. Among 289 GPs, only 120 met eligible criteria for quality assessment of data collection during the study period. Data quality and completeness have been already validated in previous drug-utilization studies, ensuring that Arianna database is an evaluable data source to estimate the effect of national and local health-policy intervention [15]–[24].

### Study population

We retrieved data on all individuals aged 15 years and over who were registered in the lists of 120 validated GPs during the years 2004–2010. Among those, we identified patients who received at least one prescription of statin during the observation years. Statin therapy could be likewise initiated by GPs or specialists (in this case passing by GP to get the prescription reimbursed by National Health System). Demographic and clinical characteristics of each user, with specific focus on statin therapy (e.g. type of statin, indications and type of prevention) and concomitant diseases were also retrieved. A treatment was defined as secondary prevention if the statin user was affected by coronary heart disease, cerebrovascular disease, peripheral arterial disease, CV procedures, or diabetes mellitus anytime prior to the prescription of statin [20].

### Study drugs

Use of the following statins was explored: simvastatin (ATC: C10AA01), lovastatin (C10AA02), pravastatin (C10AA03), fluvastatin (C10AA04), atorvastatin (C10AA05), rosuvastatin (C10AA07), and fixed combination simvastatin-ezetimibe (C10BA02).

### Data analysis

Yearly prevalence of statin treatment was measured as the ratio between the number of patients who received at least one prescription and the number of individuals alive and registered in the GPs' list in each study year. To evaluate the yearly incidence of statin use (cumulative incidence) we defined a *new user* as a patient receiving a first statin prescription within the observation year without any statin prescription recorded in the previous year. For each year, the cumulative incidence was calculated as the ratio between the number of new users and the number of individuals alive and registered in the GPs' lists, without any statin prescription in the previous year. Both, yearly- prevalence and incidence of statin use were stratified by gender and reported as rate per 1,000 inhabitants, together with 95% confidence interval (CI). Furthermore, cumulative incidence was stratified by single compound, and calendar year and characteristics of new users were evaluated according to the type of prevention.

A subgroup analysis was performed among newly initiated statin therapy with at least 6 months of follow-up to assess the adherence to the therapy at months 6, 12, 18, 24, 30, 36, 42 and 48 after start of the therapy. The Medication Possession Ratio (MPR) was calculated as the proportion of the number of pills supply dispensed over the intended period of statin treatment (in months). By assuming a single intake per day, the number of pills corresponded to the numbers of days for which the patient had been prescribed with statins [25]. For instance, in a period of 180 days, four dispensings of 30 pills' supply (i.e, 120 days) of rosuvastatin would result in an estimated MPR of 67% (120/180). MPR was categorised into 4 levels of adherence: very low (MPR≤25%), low (MPR = 26%–50%), intermediate (MPR = 50%–80%) and high adherence (MPR≥80%) [26]. Because prescriptions filled was used as a proxy for beneficiary status, users with a MPR≤25% were considered as non-adherent to statin therapy, while a MPR≥80% was established as threshold for adherence [27].

To investigate the potential predictors of non-adherence (defined as very low adherence) to statin therapy at 4-years of follow-up, we performed a sub-analysis restricted to two groups of patients: those with either very low or high levels of adherence to statin therapy at month 48.

### Statistical analysis

Chi-square for trend was used to evaluate the variation in 1-year prevalence or incidence of statin use during the observation period.

Two-tailed chi–squared test for proportion with significance level of *P*<0.05 was used for assessing the baseline characteristics of new users of different statins according to the type of prevention.

To identify predictors of non-adherence (i.e. very low adherence) to statin therapy after 4-years from the start of the treatment, univariate logistic regression model using high adherence as comparator was carried out. All the covariates significantly associated with the non-adherence in the first model have been included in the full adjusted model of multivariate logistic regression. Odds Ratios (ORs) plus CI 95% were measured for each covariate of interest.

### Ethics

Local Health Service of Caserta provided for anonymised patient records information and approved the use of data for this analysis. Data are not available for public sharing because of privacy reasons.

## Results

Out of 155,316 individuals who were registered on the GPs' lists during the observation period of seven years, we identified 15,877 patients who had received at least one prescription for statin. Among the prevalent users, we observed a similar distribution in terms of gender (8,071 women vs. 7,806 men) with a mean age of 63.7±11.5 years (data not shown).

### Yearly-prevalence and incidence of statin treatment

**Figure 1:**
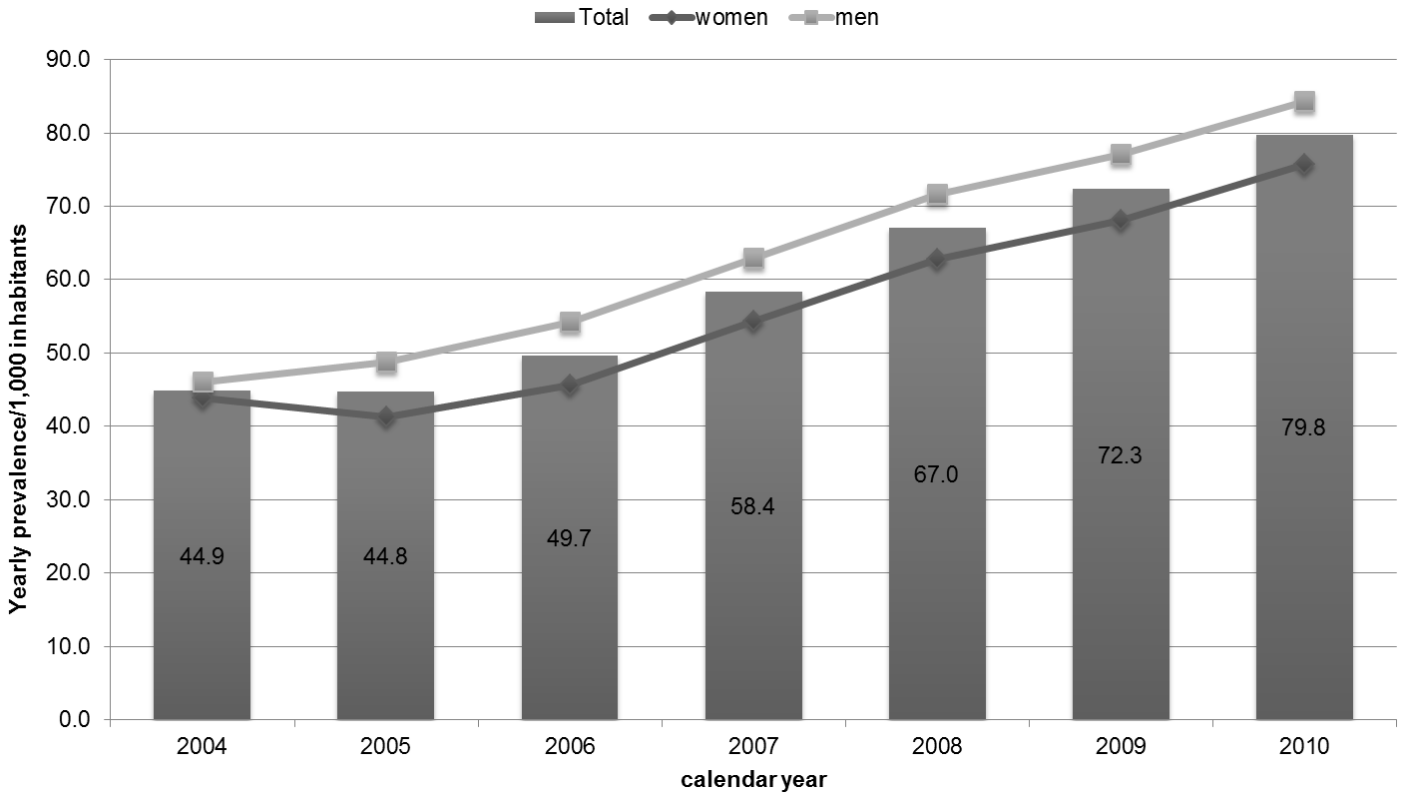
Yearly prevalence of statin use/1,000 inhabitants stratified by calendar year and gender.

Yearly-prevalence of statin use significantly increased from 44.9 (95% CI: 43.7–46.1)/1,000 inhabitants in 2004 to 79.8 (78.3–81.2) in 2010. Among women, the prevalence progressively increased from 43.9 (42.3–45.5) to 75.7 (73.7–77.6), except for a slight decrease in 2005, and among men increased linearly from 46.0 (44.3–47.7) to 84.3 (82.1–86.5). Except for 2004, the prevalence of statin use was significantly higher among men than women during the entire study period (P<0.05) (**Figure 1**).

**Figure 2:**
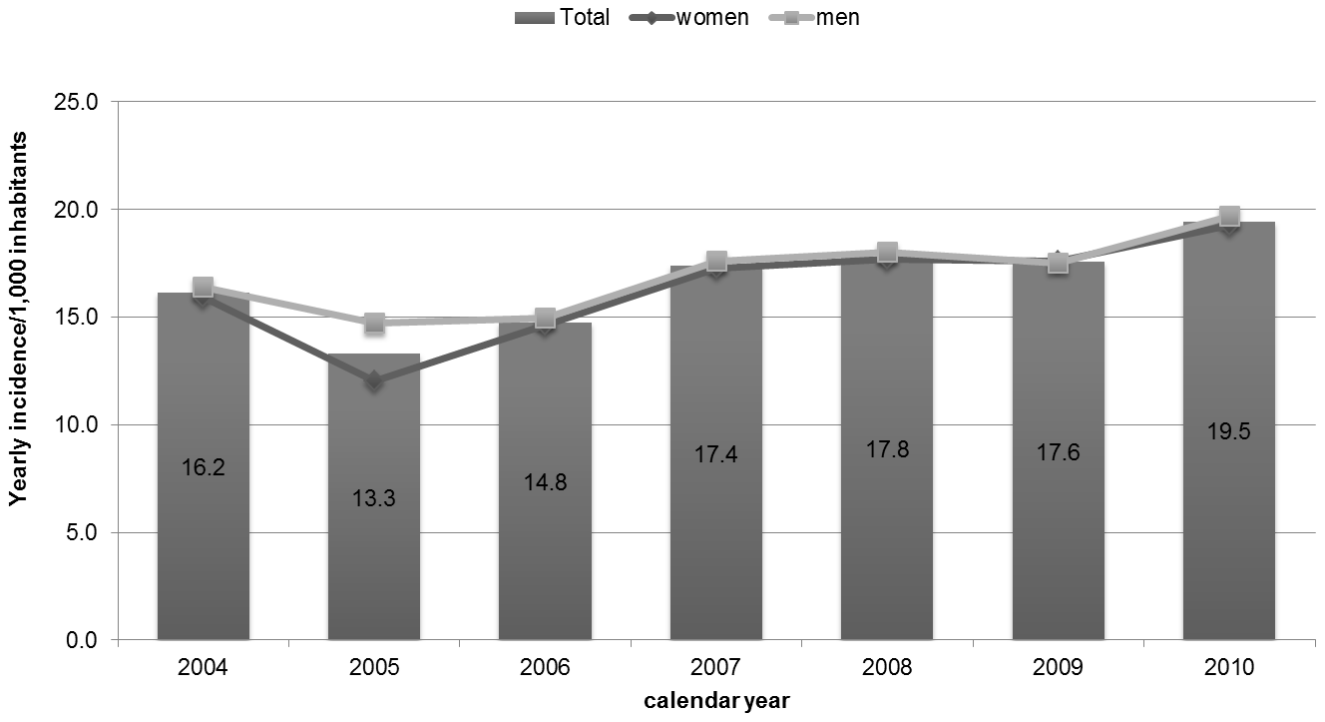
Yearly incidence of statin use/1,000 inhabitants stratified by calendar year and gender.

Yearly-incidence of statin use decreased from 16.2/1,000 (15.5–16.9) in 2004 to 13.3/1,000 (12.7–13.9) in 2005, while the situation has reversed thereafter, and the incidence rose to 19.5/1,000 (18.7–20.2) in 2010. No statistically significant differences by gender were reported among new users of statins during the study period, except in 2005 when we observed a larger reduction of the incidence in women (12.0; 11.2–12.9) than in men (14.7; 13.8–15.7) (**Figure 2**).

**Figure 3:**
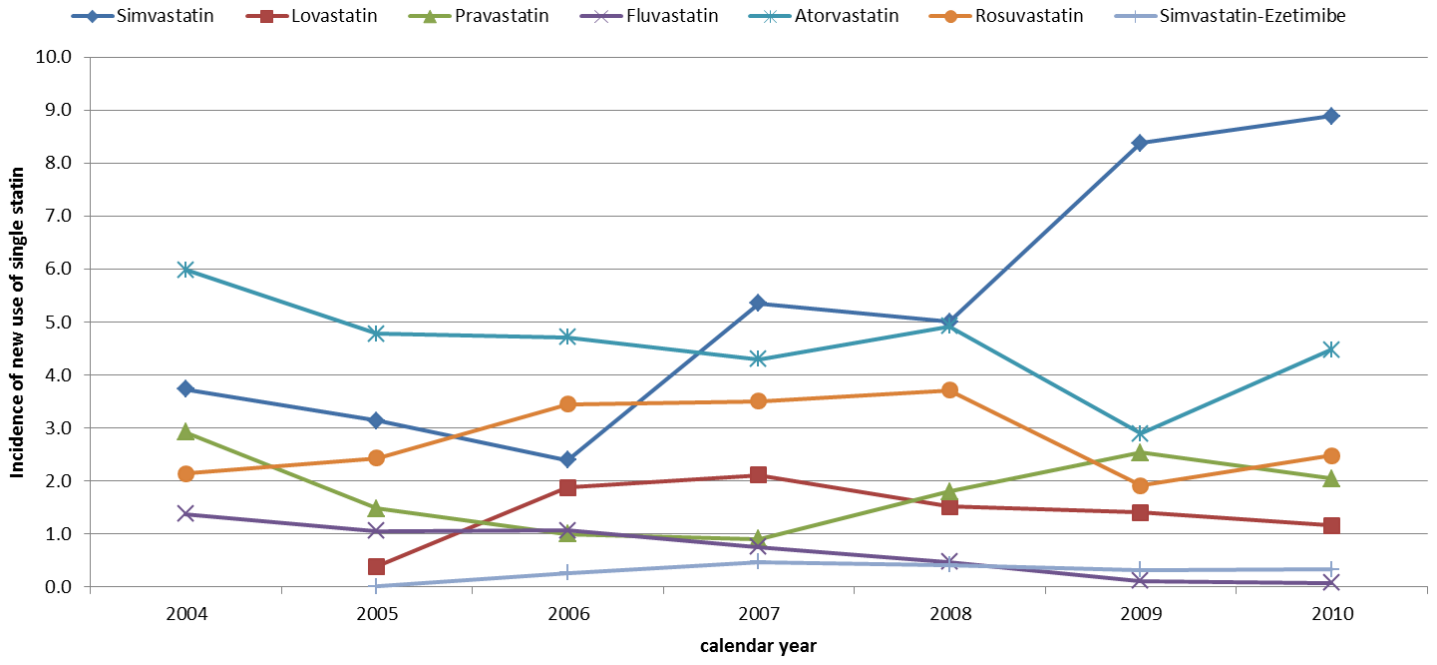
Yearly incidence of statin use stratified by individual compound.

Looking at single compounds, the incidence of atorvastatin use decreased from 6.0/1,000 (5.6-6.4) in 2004 to 4.5/1,000 (4.1–4.8) in 2010. An increased trend of new use of simvastatin was observed, with the incidence equal to 3.7/1,000 (3.4–4.1) in 2004, which was slightly decreased to 2.4/1,000 (2.1–2.7) in 2006, and increased up to 8.9/1,000 (8.4–9.4) in 2010 (**Figure 3**).

### Characteristics of new users of statins

A total of 12,980 users who newly initiated a treatment with statin were identified during the seven-years study period. Demographic and clinical characteristics of new users of statins by type of cardiovascular prevention is reported in **Table 1**. Mean age was 63.5 years (SD±11.8), with statin users treated for primary prevention who were predominantly women (57.6% vs. 42.4%, women vs. men) and significantly younger than those receiving statins for secondary prevention (60.2 vs. 65.5 years; *P*<0.01). In both types of prevention, however, women were significantly older than men (*P*<0.01).

**Table 1:** Baseline characteristics of new users of statins by type of prevention.

	Total	Primary prevention	Secondary prevention	*P value*
	*n* = 12,980 (%)	*n* = 4,811 (%)	*n* = 8,169 (%)	
**Gender**				<0.01
Women	6,673 (51.4)	2,773 (57.6)	3,900 (47.7)	
Men	6,307 (48.6)	2038 (42.4)	4,269 (52.3)	
**Mean age, y (±SD)**	63.5 (11.8)	60.2 (12.3)	65.5 (11.0)	<0.01
**Mean age by gender, y (±SD)**				<0.01
Female	65.4 (11.2)	62.2 (11.4)	67.6 (10.5)	
Male	61.5 (12.0)	57.4 (12.8)	63.5 (11.0)	
**Age category (** ***in years*** **)**				<0.01
15–44	784 (6.0)	516 (10.7)	268 (3.3)	
45–54	2,002 (15.4)	938 (19.5)	1,064 (13.0)	
55–64	3,869 (29.8)	1,533 (31.9)	2,336 (28.6)	
>65	6,325 (48.7)	1,824 (37.9)	4,501(55.1)	
**Comorbidity**				
Hyperlipidaemia	9,348 (72.0)	4,394 (91.3)	4,954 (60.6)	<0.01
Hypertension	9,207 (70.9)	2,975 (61.8)	6,232 (76.3)	<0.01
Diabetes mellitus	4,125 (31.8)	-	4,125 (50.5)	
Cardiovascular events*	3,991 (30.7)	-	3,991 (48.9)	
Other cardiovascular risk factors#	2,526 (19.5)	741 (15.4)	1,785 (21.9)	<0.01
Cerebrovascular events	1,445 (11.1)	-	1,445 (17.7)	
Peripheral arterial disease	1,189 (9.2)	-	1,189 (14.6)	
Renal failure	225 (1.7)	77 (1.6)	148 (1.8)	0.37
**Statin Type**				
Simvastatin	4,412 (34.0)	1,736 (36.1)	2,676 (32.8)	<0.01
Atorvastatin	3,457 (26.6)	1,047 (21.8)	2,410 (29.5)	<0.01
Rosuvastatin	2,001 (15.4)	750 (15.6)	1,251 (15.3)	0.67
Pravastatin	1,430 (11.0)	547 (11.4)	883 (10.8)	0.32
Lovastatin	984 (7.6)	416 (8.6)	568 (7.0)	<0.01
Fluvastatin	510 (3.9)	201 (4.2)	309 (3.8)	0.26
Simvastatin-Ezetimibe	186 (1.4)	114 (2.4)	72 (0.9)	<0.01

Values are shown as number (%) of new users, except where otherwise specified.

*Cardiovascular events include coronary heart disease, acute myocardial infarction,.

# Other cardiovascular risk factors include cardiomyopathies, valve disorders, arrhythmias, or atrial flutter/fibrillation, heart failure, or congenital heart disease.

Abbreviations: y = years; SD = Standard Deviation.

Significant differences in burden of comorbidities at time of the first prescription were observed between primary and secondary prevention statin treatment. Specifically, the proportions of hyperlipidaemia was significantly higher in primary prevention treatment than in secondary prevention (91.3% vs. 60.6% respectively; *P*<0.01). On the contrary, more patients on secondary prevention than on primary had hypertension (76.3% vs. 61.8%) and other cardiovascular risk factors (21.9% vs. 15.4%).

With regard to the specific compound, simvastatin, atorvastatin, rosuvastatin and pravastatin were the most frequently prescribed statins as first-line treatment, irrespective of the type of prevention. Atorvastatin was significantly more commonly used for secondary than primary prevention (29.5% vs 21.8%), while simvastatin, the combination simvastatin-ezetimibe and lovastatin were significantly more frequently prescribed for primary than secondary prevention (36.1% vs 32.8%; 2.4% vs 0.9%; 8.6% vs 7.0% respectively).

### Adherence to statin treatment

Among new users with at least 6 months of follow-up, the proportion of patients who had high level of adherence to statin treatment significantly decreased along 4-years of follow-up, ranging from 43.1% at month 6 of therapy to 26.1% at month 48 (**Table 2**). Predictive factors of very low adherence after 4 years from the start of the statin therapy are reported in **Table 3**. As compared to those with high adherence, patients with a very low adherence were more likely to be women (OR 1.42; CI 95% 1.24–1.62) and to be treated as primary prevention (1.84; 1.60–2.12). Consistently with the type of prevention, presence of cardiovascular or cerebrovascular diseases or diabetes mellitus was inversely associated with very low adherence. As compared to atorvastatin use, simvastatin (1.91; 1.61–2.27), pravastatin (1.79; 1.43–2.25), fluvastatin (1.41; 1.06–1.88) and lovastatin (1.77; 1.31–2.39) use was significantly associated to higher risk of very low adherence to the treatment. Full adjusted model confirmed the results from the main analysis for women, patients in primary prevention, or users of simvastatin, pravastatin, fluvastatin and lovastatin as predictive factors of non-adherence (**Table 3**).

**Table 2:** Adherence level to new treatment with any statin at different time period of follow-up.

*Follow-up (in months)*	*Total new users* *	*Adherence level* #			
		Very low (MPR≤25%)	Low (MPR = 26–50%)	Intermediate (MPR = 50–80%)	High (MPR≥80%)
		n (%)	n (%)	n (%)	n (%)
**6**	12,235	2,531 (20.7)	2,329 (19.0)	2,099 (17.2)	5,276 (43.1)
**12**	11,081	3,380 (30.5)	1,649 (14.9)	1,998 (18.0)	4,054 (36.6)
**18**	10,232	3,292 (32.2)	1,578 (15.4)	1,931 (18.9)	3,431 (33.5)
**24**	9,352	3,244 (34.7)	1,321 (14.1)	1,838 (19.7)	2,949 (31.5)
**30**	8,554	3,019 (35.3)	1,274 (14.9)	1,692 (19.8)	2,569 (30.0)
**36**	7,527	2,665 (35.4)	1,221 (16.2)	1,488 (19.8)	2,153 (28.6)
**42**	6,644	2,399 (36.1)	1,130 (17.0)	1,250 (18.8)	1,865 (28.1)
**48**	5,749	2,178 (37.9)	973 (16.9)	1,097 (19.1)	1,501 (26.1)

*only new users with at least 6 months of follow-up have been included in this analysis.

# adherence to therapy was assessed by MPR (Medical Possession Ratio) as the ratio between the number of pills supply of medication dispensed and the intended period of statin treatment (in months). This indicator was categorised into 4 coverage groups: very low (MPR ≤25%); low (MPR = 26%–50%); intermediate (MPR = 50–80%); High (MPR≥80%).

**Table 3:** Predictive factors at 4-year follow-up of very low adherent (*n* = 2,178) compared to high adherent (*n* = 1,501) to the statin treatment.

	Very low adherent*	High adherent*	OR& (95% CI)	P value	OR_adjusted_ &(95% CI)	P value
	*n* = 2,178 (%)	*n* = 1,501 (%)				
**Gender**						
Men	1,005 *(46.1)*	824 *(54.9)*	ref.		ref.	
Women	1,173 *(53.9)*	677 *(45.1)*	1.42 (1.24–1.62)	<0.01	1.26 (1.10–1.45)	<0.01
**Prevention type**						
Secondary	1,244 (57.1)	1,066 (71.0)	ref.		ref.	
Primary	934 (42.9)	435 (29.0)	1.84 (1.60–2.12)	<0.01	1.64 (1.29–2.07)	<0.01
**Comorbidity history** $						
Hyperlipidaemia	1743 (80.0)	981 (65.4)	2.12 (1.83–2.47)	<0.01	1.79 (1.53–2.11)	<0.01
Hypertension	1518 (69.7)	1029 (68.6)	1.05 (0.91–1.21)	0.46	-	
Diabetes mellitus	649 (29.8)	495 (33.0)	0.86 (0.75–0.99)	0.04	1.14 (0.94–1.37)	0.18
Cardiovascular events	642 (29.5)	599 (39.9)	0.63 (0.55–0.72)	<0.01	0.99 (0.82–1.20)	0.91
Other cardiovascular risk factors#	414 (19.0)	263 (17.5)	1.10 (0.93–1.31)	0.25	—	
Cerebrovascular events	212 (9.7)	182 (12.1)	0.78 (0.63–0.95)	0.02	0.99 (0.78–1.26)	0.90
Peripheral arterial disease	189 (8.7)	136 (9.1)	0.95 (0.76–1.20)	0.69	-	
Renal failure	42 (1.9)	19 (1.3)	1.53 (0.89–2.65)	0.13	-	
**Statin therapy**						
Atorvastatin▾	631 (29.0)	591 (39.4)	ref.		ref.	
Simvastatin	677 (31.1)	332 (22.1)	1.91 (1.61–2.27)	<0.01	1.92 (1.63–2.27)	<0.01
Rosuvastatin	281 (12.9)	241 (16.1)	1.09 (0.89–1.34)	0.40	-	
Pravastatin	293 (13.5)	153 (10.2)	1.79 (1.43–2.25)	<0.01	1.77 (1.42–2.21)	<0.01
Fluvastatin	142 (6.5)	94 (6.3)	1.41 (1.06–1.88)	0.01	1.35 (1.01–1.79)	0.04
Lovastatin	144 (6.6)	76 (5.1)	1.77 (1.31–2.39)	<0.01	1.72 (1.28–2.32)	<0.01
Simvastatin-Ezetimibe	10 (0.5)	14 (0.9)	0.67 (0.29–1.51)	0.34	-	

*Adherence level was categorized according to the MPR: very low (MPR≤25%); high (MPR≥80%).

& Odds Ratio as measure of probability of being very low adherent to the statin treatment compared the users being high adherent. All the covariates significantly associated with the non-adherence in the first model have been included in the full adjusted model.

# Other cardiovascular risk factors include cardiomyopathies, valve disorders, arrhythmias, or atrial flutter/fibrillation, heart failure, or congenital heart disease.

$ Absence of comorbidity as reference.

▾ Users of atorvastatin have been defined as reference group because atorvastatin resulted the most prescribed statins in this specific subcohort of analysis and, overall, in Italy, as reported in OSMED; in addition, the choice is consistent with a previous published study on predictors of adherence [3], [28], [36], [37]

Abbreviations: MPR: Medication Possession Ratio; OR = Odds Ratio; CI = confidence interval.

## Discussion

Our population-based drug-utilization study focused on the change in the GPs' behaviour on prescribing patter of statin in a primary care database of Southern Italy according to the health policy interventions, both national and regional. Moreover, the results of this study identified several factors as predictors of non-adherence to the treatment, that is generally frequent in newly prescribed patients [28].

As expected, yearly-prevalence of statin use has almost doubled from 2004 to 2010. This is in accordance to previous studies that found a constantly growing use of statins in most European countries since their marketing [29], [30], including Italy [3], [31]–[37]. Several studies explored the prescribing pattern of statins in different Italian regional settings [19], [38]. In particular, a study performed in Northern Italy over a 10-year period (1994–2003) reported a 28% average increase per year [39]. As well-documented, the rapid increase of statin use is attributable to several factors, including the rising awareness of the evidence-based effectiveness of these drugs, the government policies promoting more aggressive management of cardiovascular risk factors, and an increase of life expectancy in patients with CVD.

Despite of this overall increasing trend, we observed no change of prevalence in 2005. This result is in line with the health policy intervention issued on November 2004 by AIFA that revised the reimbursement criteria of statins by introducing the evaluation of cardiovascular risk charts in the management of dyslipidaemia [5], [40]. The impact of this regulatory action was confirmed in our analysis of the yearly-incidence of statin use, which significantly decreased in 2005, specifically for women. Indeed, these risk charts led to a reduction in statin use according to the fact that Italian population is thought to have relatively lower cardiovascular mortality than other countries [29]. Moreover, this reduction was mainly related to women on the basis of scientific evidence supporting that cardiovascular risk factors affect more men than women [41], [42]. This peculiar trend was already reported in a study that explored statin utilization in the same setting but over a shorter period of observation (2003–2005) [19]. Because our data cover a longer period of time, we were able to analyze the long-term effect of this intervention, in terms of management of dyslipidaemic patients according to these new cardiovascular risk charts. Thus, as expected, the incidence of statin use progressively rose from 2006 until 2008, year of the disclosure of new regional policy intervention in Campania Region [6]. The *Delibera Regionale*, in order to stimulate diagnostic and therapeutic appropriateness in terms of cost-efficacy, stated that the prescription of statins in new users should be considered only after three months-period of diet, physical activity or smoke discontinuation. In addition, when starting a new treatment, the use of one the two free of patent statins, simvastatin and pravastatin, should be preferred in patients with high cardiovascular risk [6]. The impact of this policy intervention is reflected on no variation in the yearly-incidence of overall statin use observed over two years 2008–2009, followed by an increase thereafter. Moreover, this influence was even more evident in terms of single statin use. Interestingly, as prompt response from GPs to the regional policy, the incidence of use per 1,000 inhabitants on 2009 drastically decreased for atorvastatin and rosuvastatin while increased for simvastatin and pravastatin.

According to the type of prevention, although the point prevalence and incidence of statin use were lower for women than for men, there were more women than men in terms of the absolute number of newly initiated treatment anytime in our observation period. Surprisingly, the proportion of women was higher in primary than in secondary prevention. This is noteworthy because current evidence of lipid-lowering from clinical trials showing that women are relatively protected from cardiovascular events until menopausal age, support the use of statins on secondary prevention in women with previous coronary diseases [43], [44]. Nevertheless, since some recent evidence showed similar results to our study [45], it could imply that the focus on cardiovascular risk treatment in women is rising in the most recent years [46]. However, in terms of age, our results showing that women that newly initiated a statin medication are significantly older than men are consistent with previous investigations [44].

Looking at comorbidity history, we observed several differences among people starting on primary or secondary prevention, with more individuals with hyperlipidaemia or hypertension on primary prevention treatment and more patients with other cardiovascular risk factors, like arrhythmias valves disorders, cardiomyopathies or heart failure on secondary prevention treatment.

With regard to individual medications, simvastatin, atorvastatin, rosuvastatin and pravastatin, were the most frequently prescribed statins as a first-line treatment, irrespective of the type of prevention, in line with previous investigations [19], [30], but in contrast with the latest Italian National Reports of medication use (OSMED) which identified atorvastatin as the most prescribed statin in Italy, followed by rosuvastatin and simvastatin [3], [36], [37]. The discrepancies across national and regional settings could be explained by the clinical impact of the regional policy intervention promoting the use of statins free of patent, as simvastatin and pravastatin. This result is, as described above, more evident from the analysis of the incidence stratified by calendar year and molecules, which showed an increase of new use of these two statins after 2008. On the other hand, the heterogeneity between results from our study and from OSMED could be due to different methodological measure of prescriptions as OSMED explored prevalent and naive users of statins while we focused only on naive users [3], [31]–[37].

Stratifying by the type of prevention, atorvastatin was significantly more prescribed for secondary prevention than for primary. A recent meta-analysis on comparative benefits of statins on major cerebrovascular events suggested that, although any statin therapy is associated with a significant reduction in cerebrovascular events in secondary prevention, only atorvastatin resulted in significantly fewer events than controls [47]. On the contrary, simvastatin and lovastatin were prescribed mainly for primary prevention. Nevertheless, there are no recommendations supporting the preferential use of a particular statin for primary or secondary prevention [48].

Despite several randomized controlled clinical trials have shown that only a continuous treatment with statins is effective in achieving a reduction of cardiovascular morbidity and mortality, as pointed out by the *Delibera Regionale*, we found that less than 50% of patients who newly initiated a statin were still adherent to the treatment (with MPR≥80%) after six months of follow-up, with a further reduction to 26% after 4-year of follow-up. This data are consistent with previous investigations that suggested a poor level of adherence to statin treatment and, consecutively, a reduction of long-term effectiveness. Looking at the predictive factors of non-adherence after 4-years from the start of the statin therapy, the probability to be non-adherent was 26% higher for women than for men, suggesting that women have lower perceived risk of disease. Moreover, patients on primary prevention had 64% higher probability to be non-adherent than those on secondary. In line with previous studies performed in similar Italian settings, our data suggested that starting a statin for secondary prevention seems to be predictive factor for higher long-term adherence. This could be explained by the hypothesis that, while the healthiest people has a minor perception of the risk, less-healthy people (i.e. patients with history of CVD) are strongly motivated, by GPs or by themselves, to continue the therapy to achieve the therapeutic goal [49], [50]. In addition, the use of simvastatin, pravastatin, fluvastatin or lovastatin as first line therapy seems to predict higher probability to be non-adherent as compared to atorvastatin. There is no previous evidence to explain these results; the higher use of atorvastatin in secondary prevention could partially explain this evidence. Moreover, since the discontinuation of treatment, as defined for MPR calculation, does not consider if a patient switched across statins, we cannot exclude that people starting with simvastatin, pravastatin, fluvastatin or lovastatin could have changed the type of statin as consequence of the occurrence of adverse effects or as lack of efficacy. To date, lack of efficacy and drug therapeutic failure (DTF) are included within the wider definition of adverse drug event given by the World Health Organisation (WHO) and it is proposed as a peculiar type of adverse drug reaction designated as “Failure” [51]–[53]. However, since healthcare providers may have a crucial impact on patients' adherence to medication, the identification of these patient-related predictors for non-adherence could be useful to increase the compliance.

### Strengths and limitations

This drug-utilization study aimed to measure the use of statin in a general practice of southern Italy. The availability of prescription data over a seven-year period (2004–2010) allowed us to evaluate the implications of nationwide and regionwide health policy interventions in the real clinical practice. Finally, very long observation period and availability of many variables routinely collected through this healthcare record database provide insight into long-term adherence to statin therapy. Several limitations of our study warrant caution. First of all, we used outpatient prescription data, and we had no information whether prescriptions were actually filled. Nevertheless, since our study was aimed to evaluate the GPs' prescribing behaviour according to health policy interventions, this prescription database can be considered suitable for such investigation. However, only less than half of all the GPs enrolled in the Arianna database previously trained in data collection techniques. This could represent a potential information bias reducing the sensitivity to investigate the prescribing trend of statins. On the other hand, the accuracy of these high-qualified GPs ensured an high specificity in order to explore the predictive factors of non-adherence, likewise to previously published studies showing that the Arianna database provides accurate and reliable information on drug utilization and is an evaluable data source to estimate the effect of national and local health-policy intervention [15]–[18]. Second, our data did not allow us to explore other potential predictors of non-adherence, such as smoking, overweight/obesity, cholesterolemia, liver enzymes or Creatine Phosphokinase levels, which are not routinely collected in the database. Finally, we used Medication Possession Ratio (MPR) as an adherence measure. Discontinuation might fulfil only part of the full definition of non-adherence, which, moreover, includes very low adherence, intermittent use and switch between statins.

### Conclusions

The increase prevalence and incidence of statin use across 2004–2010 in Southern Italy was consistent with the trend observed in other national and regional settings, as validation of such database for observational studies. Moreover, the identification of several patient-related predictors for non-adherence (such as gender and primary prevention) to the treatment could be suitable for GPs to improve their patients' behaviours. These data provide evidence for a good general practice in Southern Italy according to the health policy interventions either national or regional, issued to promote the appropriateness of the statin prescription. However, on 2011 AIFA revised again the statin reimbursement criteria (NOTA 13) in light of updated scientific evidence. Further long-term follow-up studies, starting from 2012, need to be performed to explore the long-term clinical impact of this intervention.
