# Correction: Pattern of Statin Use in Southern Italian Primary Care: Can Prescription Databases Be Used for Monitoring Long-Term Adherence to the Treatment?

**DOI:** 10.1371/journal.pone.0112037

**Published:** 2014-10-22

**Authors:** The PLOS ONE Staff

The affiliation for the tenth author is incorrect. Achille Patrizio Caputi is not affiliated with #2 but with #3 Clinical and Experimental Medicine Department, University of Messina, Messina, Italy.
